# The impact of maternal vulnerability on stress biomarkers and first-trimester growth: the Rotterdam Periconceptional Cohort (Predict Study)

**DOI:** 10.1093/humrep/deae211

**Published:** 2024-09-19

**Authors:** Sofie K M Van Zundert, Lenie Van Rossem, Mina Mirzaian, Sten P Willemsen, Lotte W Voskamp, Wietske A P Bastiaansen, Darya Nikpayam, Pieter H Griffioen, Wim F Schilleman, Anton H J Koning, Sjoerd A A Van Den Berg, Melek Rousian, Ron H N Van Schaik, Régine P M Steegers-Theunissen

**Affiliations:** Department of Obstetrics and Gynecology, Erasmus MC, University Medical Center, Rotterdam, the Netherlands; Department of Clinical Chemistry, Erasmus MC, University Medical Center, Rotterdam, the Netherlands; Department of Obstetrics and Gynecology, Erasmus MC, University Medical Center, Rotterdam, the Netherlands; Department of Clinical Chemistry, Erasmus MC, University Medical Center, Rotterdam, the Netherlands; Department of Obstetrics and Gynecology, Erasmus MC, University Medical Center, Rotterdam, the Netherlands; Department of Biostatistics, Erasmus MC, University Medical Center, Rotterdam, the Netherlands; Department of Obstetrics and Gynecology, Erasmus MC, University Medical Center, Rotterdam, the Netherlands; Division of Pharmacology and Vascular Medicine, Department of Internal Medicine, Erasmus MC, University Medical Center, Rotterdam, the Netherlands; Department of Obstetrics and Gynecology, Erasmus MC, University Medical Center, Rotterdam, the Netherlands; Department of Radiology and Nuclear Medicine, Biomedical Imaging Group Rotterdam, Erasmus MC, University Medical Center, Rotterdam, the Netherlands; Department of Obstetrics and Gynecology, Erasmus MC, University Medical Center, Rotterdam, the Netherlands; Department of Clinical Chemistry, Erasmus MC, University Medical Center, Rotterdam, the Netherlands; Department of Clinical Chemistry, Erasmus MC, University Medical Center, Rotterdam, the Netherlands; Department of Pathology, Erasmus MC, University Medical Center, Rotterdam, the Netherlands; Department of Clinical Chemistry, Erasmus MC, University Medical Center, Rotterdam, the Netherlands; Department of Internal Medicine, Erasmus MC, University Medical Center, Rotterdam, the Netherlands; Department of Obstetrics and Gynecology, Erasmus MC, University Medical Center, Rotterdam, the Netherlands; Department of Clinical Chemistry, Erasmus MC, University Medical Center, Rotterdam, the Netherlands; Department of Obstetrics and Gynecology, Erasmus MC, University Medical Center, Rotterdam, the Netherlands

**Keywords:** periconceptional vulnerability, pregnancy, stress pathways, hair cortisol, hair cortisone, tryptophan, embryo

## Abstract

**STUDY QUESTION:**

Is the degree of maternal vulnerability positively associated with stress biomarkers (stress hormones, C-reactive protein, tryptophan metabolites, and one-carbon metabolites), and does long-term exposure to stress hormones reduce first-trimester growth?

**SUMMARY ANSWER:**

The maternal vulnerability risk score is positively associated with concentrations of hair cortisol and cortisone and negatively with tryptophan, while higher hair cortisol concentrations are associated with reduced first-trimester growth without mediation of tryptophan.

**WHAT IS KNOWN ALREADY:**

A high degree of maternal vulnerability during the periconception period is associated with impaired first-trimester growth and pregnancy complications, with consequences for long-term health of the child and future life course. However, due to the challenges of early identification of vulnerable women, the uptake of periconception care is low in this target group.

**STUDY DESIGN, SIZE, DURATION:**

Between June 2022 and June 2023, this study was conducted in a sub-cohort of 160 pregnant women participating in the Rotterdam Periconceptional Cohort (Predict Study), an ongoing prospective tertiary hospital-based cohort.

**PARTICIPANTS/MATERIALS, SETTING, METHODS:**

One hundred and thirty-two women with ongoing pregnancies and available stress biomarker data were included in the analysis. Data on periconceptional social, lifestyle, and medical risk factors were collected via self-administered questionnaires, and these factors were used for the development of a composite maternal vulnerability risk score. Stress biomarkers, including stress hormones (hair cortisol and cortisone) and inflammatory and oxidative stress biomarkers (C-reactive protein, total homocysteine, and tryptophan metabolites) were determined in the first trimester of pregnancy. First-trimester growth was assessed by crown–rump length (CRL) and embryonic volume (EV) measurements at 7, 9, and 11 weeks gestation by making use of an artificial intelligence algorithm and virtual reality techniques using 3D ultrasound data sets. The associations between the maternal vulnerability risk score and stress biomarkers were identified using linear regression models, and between stress hormones and CRL- and EV-trajectories using mixed models. A mediation analysis was performed to assess the contribution of tryptophan. All associations were adjusted for potential confounders, which were identified using a data-driven approach. Several sensitivity analyses were performed to check the robustness of the findings.

**MAIN RESULTS AND THE ROLE OF CHANCE:**

The maternal vulnerability risk score was positively associated with concentrations of hair cortisol and cortisone (pg/mg) (β = 0.366, 95% CI = 0.010–0.722; β = 0.897, 95% CI = 0.102–1.691, respectively), and negatively with tryptophan concentrations (µmol/L) (β = –1.637, 95% CI = –2.693 to –0.582). No associations revealed for C-reactive protein and total homocysteine. Higher hair cortisol concentrations were associated with reduced EV-trajectories (^3^√EV: β = –0.010, 95% CI = –0.017 to –0.002), while no associations were found with CRL-trajectories. Mediation by tryptophan was not shown.

**LIMITATIONS, REASONS FOR CAUTION:**

Residual confounding cannot be ruled out, and the external validity may be limited due to the study’s single-center observational design in a tertiary hospital.

**WIDER IMPLICATIONS OF THE FINDINGS:**

There is mounting evidence that a high degree of maternal vulnerability negatively affects maternal and perinatal health, and that of the future life course. The results of our study emphasize the need to identify highly vulnerable women as early as possible, at least before conception. Our findings suggest that the chronic stress response and alterations of the maternal tryptophan metabolism are involved in maternal vulnerability, affecting first-trimester growth, with potential impact on the long-term health of the offspring.

**STUDY FUNDING/COMPETING INTEREST(S):**

This study was funded by the Departments of Obstetrics and Gynecology and Clinical Chemistry of the Erasmus MC, University Medical Center, Rotterdam, the Netherlands, and the Junior Award granted by the De Snoo—van ’t Hoogerhuijs Foundation in March 2022. There are no conflicts of interest.

**TRIAL REGISTRATION NUMBER:**

N/A.

## Introduction

Women are considered ‘vulnerable’ when social, lifestyle, or medical risk factors, such as residing in a deprived neighborhood, smoking, or having a chronic disease, impair the women’s health, with potential implications for their offspring (van der Meer *et al.*, 2020). Exposure to these adversities during the periconception period, which covers the 14 weeks before conception and the 10 weeks thereafter, can have lifelong and transgenerational effects through epigenetic mechanisms, as proposed by the Developmental Origins of Health and Disease paradigm (Barker, 2007; Steegers-Theunissen *et al.*, 2013). In a previous study of our research group, we have shown that a higher degree of periconceptional maternal vulnerability, determined by the number of accumulating risk factors, is associated with impaired first-trimester growth (van Zundert *et al.*, 2022). Hence, early identification of highly vulnerable women is crucial with regards to prevention and early interventions (van Uitert *et al.*, 2013; Jaddoe *et al.*, 2014). Gaining more insight into the biological pathways involved in maternal vulnerability is the first step in uncovering the mechanisms that underlie the negative association between maternal vulnerability and first-trimester growth.

Exposure to adversities can disrupt homeostasis in cells and tissues, which engages a stress response. A key player in the stress response is activation of the hypothalamic–pituitary–adrenal (HPA) axis, with cortisol as end product. Prolongation of this stress response (chronic stress) by accumulated exposure to adversities leads to long-term increased cortisol concentrations (Russell and Lightman, 2019). Animal studies have shown that physiological concentrations of cortisol are required for cell differentiation and maturation of tissues and organ systems, but that excessive cortisol concentrations can harm gametogenesis, and embryonic and placental development (Cottrell and Seckl, 2009; Joseph and Whirledge, 2017). Even though cortisol concentrations rise during pregnancy as a result of the placenta releasing corticotrophin-releasing hormone, the HPA axis remains responsive to stress (Giesbrecht *et al.*, 2013).

Chronic stress can induce inflammation and oxidative stress and dysregulate metabolic pathways, including tryptophan metabolism (Black and Garbutt, 2002; Russell and Lightman, 2019). Under physiological conditions, 95% of tryptophan is metabolized via the kynurenine pathway, and 1–5% through the serotonin pathway (Supplementary Fig. S1), with the remainder used for the indole pathway and protein synthesis (Bender, 1983; Oxenkrug, 2010). Metabolites of the kynurenine and serotonin pathways are required for many important processes during the periconception period, including immune regulation, anti-oxidative processes, and regulation of vascular tone (Badawy, 2015; Badawy, 2017; Broekhuizen *et al.*, 2021). In addition, it provides methyl donors and shares substrates and cofactors with the one-carbon metabolism. The one-carbon metabolism is essential in providing one-carbon moieties for cell multiplication, cell differentiation, and epigenetic programming (Steegers-Theunissen *et al.*, 2013; Greenberg and Bourc'his, 2019). Homocysteine is a sensitive marker of derangements of the one-carbon metabolism, and is involved in oxidative stress (Steegers-Theunissen *et al.*, 2013).

**Figure 1.**
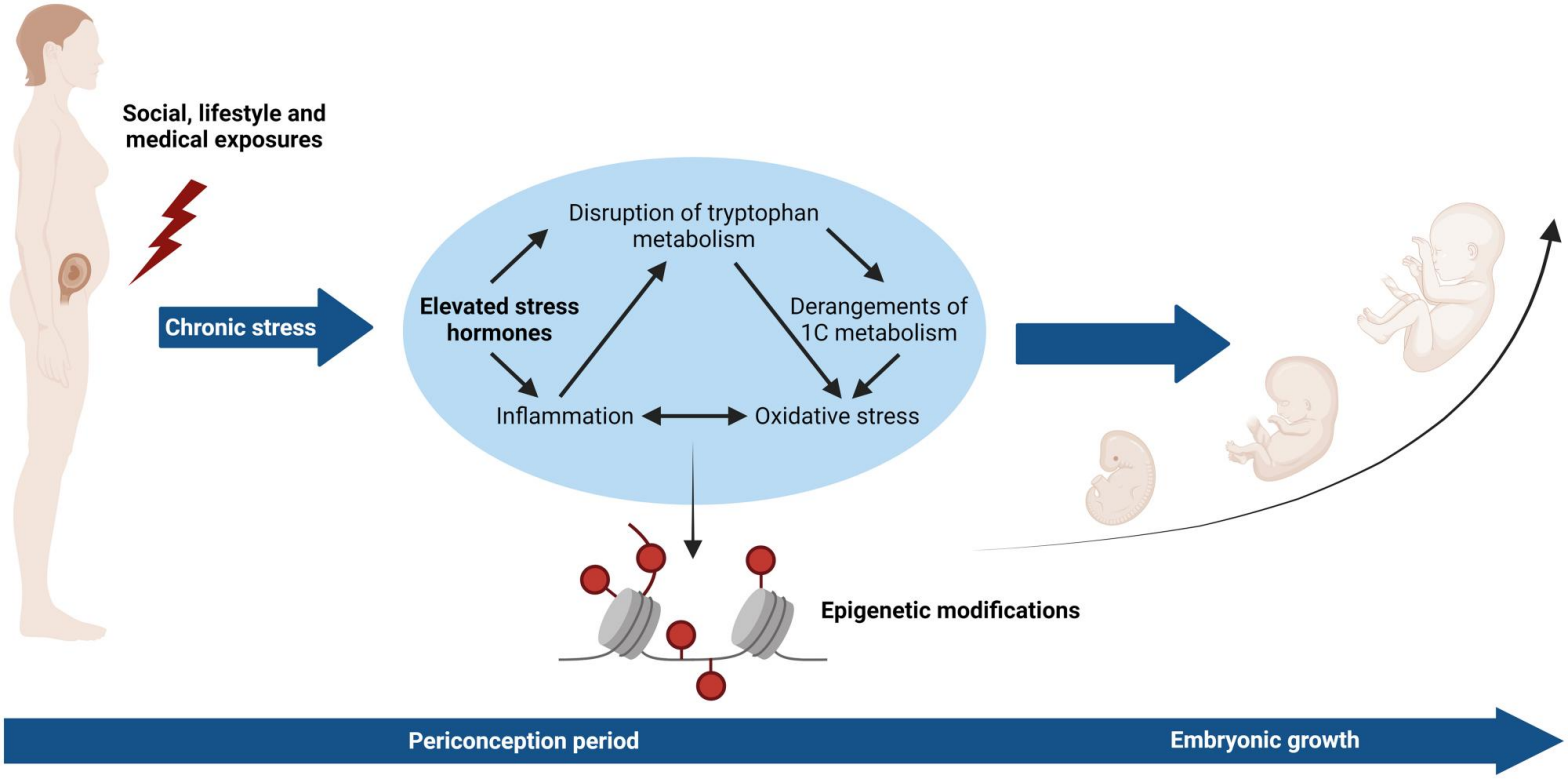
**Biological pathways potentially involved in maternal vulnerability**.

Our hypothesis is that a higher degree of maternal vulnerability is associated with chronic stress, increased concentrations of stress hormones (hair cortisol and cortisone) and inflammatory and oxidative stress markers (C-reactive protein (CRP) and total homocysteine), and reduced concentrations of tryptophan metabolites (tryptophan, kynurenine, and serotonin pathway metabolites including 5-hydroxytryptophan, 5-hydroxytryptamine, and 5-hydroxyindole acetic acid) (Fig. 1). Therefore, the main objective of this study is to explore whether a higher degree of maternal vulnerability during the periconception period is positively associated with stress biomarkers. The secondary objective of this explorative study is to investigate whether the concentrations of stress hormones in the first trimester of pregnancy are negatively associated with first-trimester growth, and mediated by inflammatory and oxidative stress biomarkers.

## Materials and methods

### Study design and setting

This study was conducted in a sub-cohort of the Rotterdam Periconceptional Cohort (Predict Study) between June 2022 and June 2023. The Predict Study is an ongoing prospective tertiary hospital-based cohort embedded in the outpatient clinic of the Department of Obstetrics and Gynecology of the Erasmus MC, Rotterdam, the Netherlands (Steegers-Theunissen *et al.*, 2016; Rousian *et al.*, 2021). Women aged 18 years or older, with proficiency in speaking and reading the Dutch language, and a singleton pregnancy of <10 weeks gestation were eligible for participation.

### Study population

**Figure 2.**
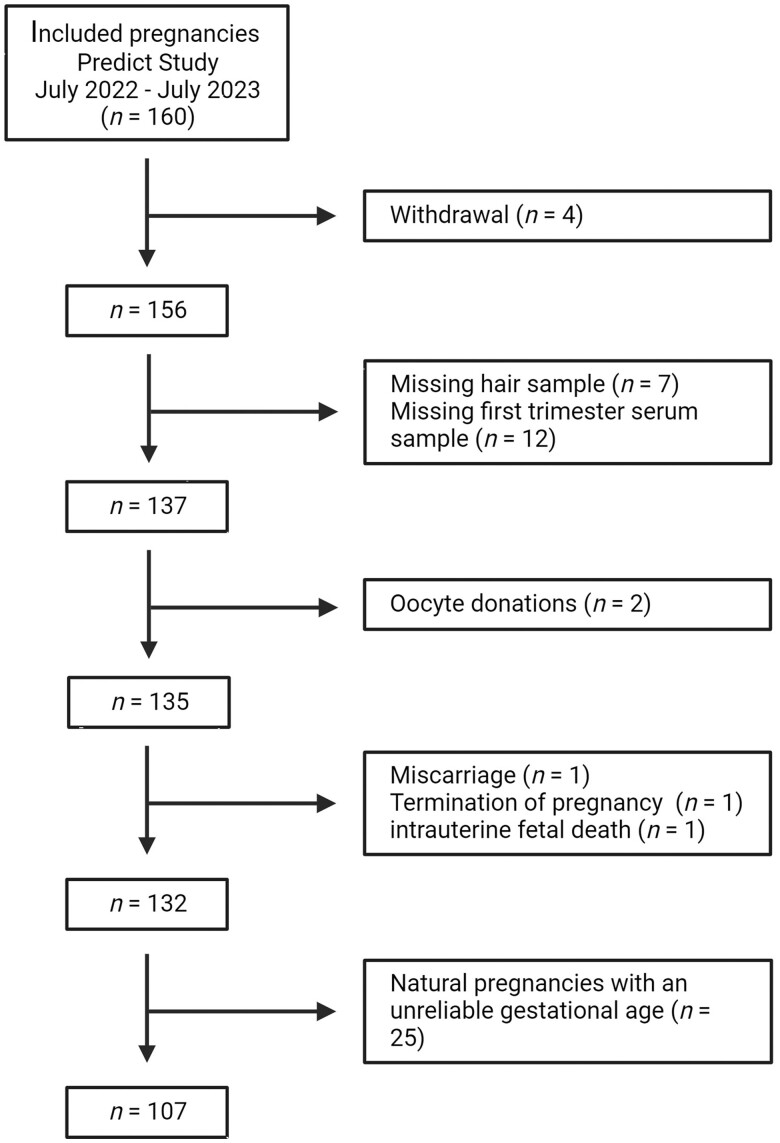
**Flowchart of the study population**.

Over a 1-year period, 160 pregnant women were enrolled in this sub-cohort (Fig. 2). Women who withdrew from the study (n* *=* *4), did not provide a hair sample (n* *=* *7) or blood sample (n* *=* *12) were excluded. Women who conceived following oocyte donation (n* *=* *2) were excluded as there was no data available of the donors. Pregnancies with an increased risk of impaired first-trimester growth, e.g. miscarriages (n* *=* *1), terminated pregnancies with a congenital anomaly (n* *=* *1), and intrauterine fetal deaths (n* *=* *1) were excluded. This resulted in a total study population of 132 women.

Gestational age was calculated from the conception date. For pregnancies conceived after IVF or ICSI treatment, the gestational age was calculated from the oocyte retrieval date adding 14 days. If a cryopreserved embryo was used, the gestational age was calculated from the embryo transfer date adding 14 days and additionally adding the number of days the embryo developed before cryopreservation (5 or 6 days). For naturally conceived pregnancies in women with a regular menstrual cycle, the gestational age was calculated from the first day of the last menstrual period (LMP) and was adjusted for the duration of the menstrual cycle when the menstrual cycle deviated >3 days from 28 days.

The gestational age was considered unreliable if the LMP or conception date was missing or women had an irregular cycle or when the gestational age calculated from the LMP differed ≥7 days from the gestational age based on the crown–rump length (CRL) at the 9th week ultrasound scan. Since gestational age is the most important determinant of first-trimester growth, these pregnancies (n* *=* *25) were excluded from the main analysis with first-trimester growth as outcome.

### Data collection

#### Stress biomarkers

Non-fasting blood samples were collected at 10.5 (SD = 1.6) weeks gestation. Tryptophan metabolites, including tryptophan, kynurenine, 5-hydroxytryptophan, 5-hydroxytryptamine, and 5-hydroxyindole acetic acid, were measured in serum using a validated liquid chromatography tandem mass spectrometry (LC-MS/MS) method (van Zundert *et al.*, 2023). This study focused on tryptophan metabolites of the kynurenine and serotonin pathways, which account for 95% and 1–5% of tryptophan metabolism, rather than on the indole pathway (Badawy, 2015). Total homocysteine was measured in EDTA plasma using LC-MS/MS. CRP was measured in serum using an immuno-turbidimetric assay (Roche CRP4 on C502, Mannheim, Germany).

Hair samples were collected during the intake appointment in the hospital at 8.9 (SD = 2.3) weeks gestation. A hair strand of ∼100–200 hairs was cut from the posterior vertex as close to the scalp as possible. The most proximal 1-cm hair, which is thought to reflect the cortisol and cortisone exposure during the previous month, was used for determination of hair cortisol and cortisone. A comprehensive description of the LC-MS/MS method has been published recently (Mirzaian *et al.*, 2023). Supplementary Table S1 summarizes the stress biomarker distribution and quantification of the study samples.

#### Maternal vulnerability risk score

In accordance with our earlier study, we selected 14 risk factors, also called vulnerability markers, and grouped them into three domains: social (n = 5), lifestyle (n = 5), and medical (n = 4) (Supplementary Table S2). The degree of periconceptional maternal vulnerability was assessed by creating a composite maternal vulnerability risk score, which consisted of the number of risk factors women were exposed to during the periconception period (van der Meer *et al.*, 2020; van Zundert *et al.*, 2022).

Anthropometric measurements were performed by a trained research nurse using a standardized protocol during the intake appointment in the hospital. Furthermore, women filled out four questionnaires during the first trimester of pregnancy before this intake appointment, including a general questionnaire, a Food Frequency Questionnaire (FFQ), the Edinburgh Postnatal (Postpartum) Depression Scale (EPDS), and the Pittsburgh Sleep Quality Index (PSQI). The general questionnaire covered maternal characteristics and lifestyle behaviors during the periconception period. The validated FFQ was used to assess dietary intake of the previous month (Feunekes *et al.*, 1993; Verkleij-Hagoort *et al.*, 2007; Fayet *et al.*, 2011). The 10-question EPDS is a reliable instrument for screening depression, with a score ranging from 0 to 30, where a score ≥10 indicates a possible depression (Cox *et al.*, 1987; Levis *et al.*, 2020). The validated 19-question PSQI assesses sleep quality of the previous month, with a score ranging from 0 to 21, and a score >5 indicating poor sleep quality (Buysse *et al.*, 1989). The EPDS and PSQI are both validated for pregnant women (Jomeen and Martin, 2007; Bergink *et al.*, 2011).

#### First-trimester growth parameters

First-trimester growth was assessed by CRL and embryonic volume (EV) trajectories during the first trimester of pregnancy. Serial standardized 3D ultrasound scans were performed by trained medical doctors at 7, 9, and 11 weeks gestation. A transvaginal (6–12 MHz) probe in combination with the GE Voluson E10 Expert System was used. The 3D ultrasound images were stored as Cartesian volumes, and the CRL was measured by a trained researcher using our developed V-Scope application on a desktop virtual reality (VR) system (Rousian *et al.*, 2011; Baken *et al.*, 2015; Rousian *et al.*, 2018). An interactive hologram was created allowing real-depth perception and thereby accurate measurements of the embryo. The CRL measurements were performed three times, and the average value was used for the analysis (Rousian *et al.*, 2010; Steegers-Theunissen *et al.*, 2016; Rousian *et al.*, 2021). The EV was measured using our recently developed and validated Artificial Intelligence (AI) algorithm (Bastiaansen *et al.*, 2023). The AI algorithm takes a 3D ultrasound data set as input and outputs binary segmentations of the embryo, which can be used to accurately measure EV. The AI algorithm has been shown to give accurate measurements comparable to our earlier semi-automatic method (intraclass correlation coefficient of 0.998) (Bastiaansen *et al.*, 2023). The results were visually checked by an expert rater using the desktop VR system (Koning *et al.*, 2009; Rousian *et al.*, 2018).

### Statistical analysis

The baseline characteristics of the total study population were described using means with SD for continuous variables and numbers with corresponding percentages for categorical variables. Biomarker concentrations were presented as medians with interquartile ranges (IQRs) considering the skewed distribution of the data. A correlation matrix was constructed using Spearman’s correlations to assess the correlation between the maternal vulnerability risk score, the EPDS score, and the PSQI score.

The associations between the maternal vulnerability risk score and biomarker concentrations were assessed using multivariable linear regression models. Model 1 was a crude model, and to improve precision, Model 2 additionally included other predictive variables that showed statistical significance (*P* ≤ 0.05) in bivariable analyses (Supplementary Table S3). For hair biomarkers, Model 2 was adjusted for corticosteroid use within the last 3 months and natural hair color. For serum biomarkers, gestational age at blood sampling was included in Model 2.

As tryptophan is an essential amino acid, we conducted a sensitivity analysis to assess whether the identified associations with tryptophan metabolites could be explained by protein intake.

The associations between stress hormones (hair cortisol and cortisone) and longitudinal CRL and EV measurements were analyzed using mixed models, taking into account the correlated repeated measurements within each pregnancy. A cubic spline function for gestational age at the 3D ultrasound scan visit and a random intercept and slope were included in all models. Model 1 was adjusted for gestational age at the 3D ultrasound scan visit. Model 2 was additionally adjusted for the previously mentioned predictors for stress hormones along with additional predictive variables for first-trimester growth parameters that had shown statistical significance (*P* ≤ 0.05) in bivariable analyses (Supplementary Table S4). As a result, Model 2 was corrected for corticosteroid use within the last 3 months, natural hair color, age, BMI, smoking, vegetable intake, and fetal sex.

A mediation analysis was performed to investigate whether other stress biomarkers associated with maternal vulnerability were part of the pathway between stress hormones and first-trimester growth. The total and direct associations were estimated, and the difference method was used to determine the indirect association. The 95% CIs of the indirect association were calculated using bootstrapping with 2000 replicates (Farland *et al.*, 2020).

Sensitivity analyses were performed to assess the robustness of the findings, including log-transformation of hair cortisol and cortisone to avoid outliers and exclusion of cases with permed hair (n = 1).

Results were displayed as effect estimates (β) with 95% CIs and corresponding *P*-values. A *P*-value ≤ 0.05 was considered statistically significant. All statistical analyses were performed using R version 4.2.1 and IBM SPSS Statistics for Windows version 28 (R Core Team, 2022).

### Ethical approval

Ethical clearance was obtained from the local Medical Ethical Committee of the Erasmus MC, University Medical Center Rotterdam (24 June 2022, Amendment A-0001, P04.1325L) and the Central Committee on Research The Hague (15 October 2004, MEC-2004-277) (Steegers-Theunissen *et al.*, 2016). All participants gave written informed consent at enrolment.

## Results

### Baseline characteristics

Table 1 shows the baseline characteristics of the study population, and the hair characteristics are presented in Supplementary Table S5. Women were on average 33.4 (SD = 4.2, range = 23.4–44.5) years at conception and had a BMI of 26.8 (SD = 4.4) kg/m^2^. Most women had a Western geographical origin (83.3%) and were highly educated (59.1%), did not smoke (90.9%), and did not consume alcohol (69.7%) nor drugs (97.7%). While more than half of the women had an inadequate intake of fruits (63.9%) and vegetables (57.1%), the majority did start using folic acid supplement use before conception (85.6%). Slightly more than half of the women conceived via IVF or ICSI (56.8%) and were nulliparous (59.8%).

**Table 1.** Baseline characteristics of the total study population.

Baseline characteristics	n = 132
Maternal general characteristics	Mean (SD)/n (%)
**Age (years)** ^a^	33.4 (4.2)
*Missing*	0
**Parity**	
Nulliparous	79 (59.8%)
Multiparous	53 (40.2%)
*Missing*	0
**BMI (kg/m²)**	26.8 (4.4)
*Missing*	0
**Geographical origin**	
Non-Western	22 (16.7%)
Western	110 (83.3%)
*Missing*	0
**Educational level**	
Low	8 (6.1%)
Medium	46 (34.8%)
High	78 (59.1%)
*Missing*	0
**Smoking**	
Yes	12 (9.1%)
No	120 (90.9%)
*Missing*	0
**Alcohol consumption**	
Yes	40 (30.3%)
No	92 (69.7%)
*Missing*	0
**Drug use**	
Yes	3 (2.3%)
No	129 (97.7%)
*Missing*	0
**Fruit intake**	
Inadequate (<2 pieces/day)	76 (63.9%)
Adequate	43 (36.1%)
*Missing*	13
**Vegetable intake**	
Inadequate (<7 days/week)	68 (57.1%)
Adequate	51 (42.9%)
*Missing*	13
**Folic acid supplement use**	
Inadequate (no or not preconception)	19 (14.4%)
Adequate	113 (85.6%)
*Missing*	0
**Mode of conception**	
IVF/ICSI	75 (56.8%)
Natural	57 (43.2%)
*Missing*	0
**Fetal sex**	
Boy	64 (50.0%)
Girl	64 (50.0%)
*Missing*	4
**Maternal vulnerability**	
**Social domain**	
Young or advanced age (<20 or ≥40 years)^a^	7 (5.3%)
*Missing*	0
Non-western geographical origin	22 (16.7%)
*Missing*	0
Single	1 (0.8%)
*Missing*	1
Deprived neighborhood (SES <–0.1)	34 (25.8%)
*Missing*	0
Low level of education (ISCED ≤2)	8 (6.1%)
*Missing*	0
**Lifestyle domain**	
Smoking, yes	12 (9.1%)
*Missing*	0
Alcohol consumption, yes	40 (30.3%)
*Missing*	0
Drug use, yes	3 (2.3%)
*Missing*	0
Inadequate fruit (<2 pieces/day) and vegetable intake (<7 days/week)	75 (63.0%)
*Missing*	13
Inadequate physical activity (<150 MPW)	90 (69.8%)
*Missing*	3
**Medical domain**	
Chronic disease (inflammatory or cardiovascular disease)	44 (33.3%)
*Missing*	0
Any medication use	71 (53.8%)
*Missing*	0
Underweight or obesity (<18.5 or ≥ 30 kg/m^2^)	26 (19.7%)
*Missing*	0
Psychiatric disorder (anxiety or depression)	26 (19.7%)
*Missing*	0
Vulnerability risk score (range = 0–14)	3.4 (1.6)
*Missing*	15
**Self-reported depression and sleep quality**	
EPDS score (range = 0–30)	5.5 (3.8)
*Missing*	3
Possible depression based on EPDS	16 (12.4%)
*Missing*	3
PSQI score (range = 0–21)	5.8 (3.5)
*Missing*	8
Poor sleep quality based on PSQI	52 (41.9%)
*Missing*	8
**First-trimester biomarker concentrations**	
Hair cortisol (pg/mg)	3.8 (2.7, 5.0)
*Missing*	5
Hair cortisone (pg/mg)	17.1 (13.1, 22.8)
*Missing*	3
Tryptophan (µmol/L)	56.0 (51.2, 63.0)
*Missing*	0
Kynurenine (µmol/L)	1.4 (1.1, 1.6)
*Missing*	0
5-Hydroxytryptophan (nmol/L)	5.7 (5.0, 6.6)
*Missing*	2
5-Hydroxytryptamine (nmol/L)	671.5 (519.5, 891.9)
*Missing*	0
5-Hydroxyindoleacetic acid (nmol/L)	46.0 (38.2, 57.7)
*Missing*	0
Total homocysteine (µmol/L)	6.0 (5.5, 7.2)
*Missing*	16
C-reactive protein (mg/L)	3.4 (2.1, 6.3)
*Missing*	0

a No age <20 years.

The continuous variables are presented as means with SD, and the categorical variables as numbers with corresponding percentages. The metabolite concentrations were presented as medians with interquartile ranges.

SES, socioeconomic status; ISCED, International Standard Classification of Education; MPW, minutes per week; EPDS, Edinburgh Postnatal (Postpartum) Depression Scale; PSQI, Pittsburgh Sleep Quality Index.

The maternal vulnerability risk score was approximately normally distributed, with a mean of 3.4 (SD = 1.6) risk factors. The mean EPDS score was 5.5 (SD = 3.8), and indicated a possible depression in 12.4% of the women. The mean PSQI score was 5.8 (SD = 3.5), indicating that the sleep quality was poor in 41.9% of the women. The correlation matrix in Supplementary Fig. S2 showed that a higher maternal vulnerability risk score was weakly correlated with more sleeping problems (*r* = 0.2) but not with depression. Besides the positive correlation between hair cortisol and cortisone concentrations (*r* = 0.6), no other strong correlations between the stress biomarkers were found.

### Stress biomarkers and maternal vulnerability

**Figure 3.**
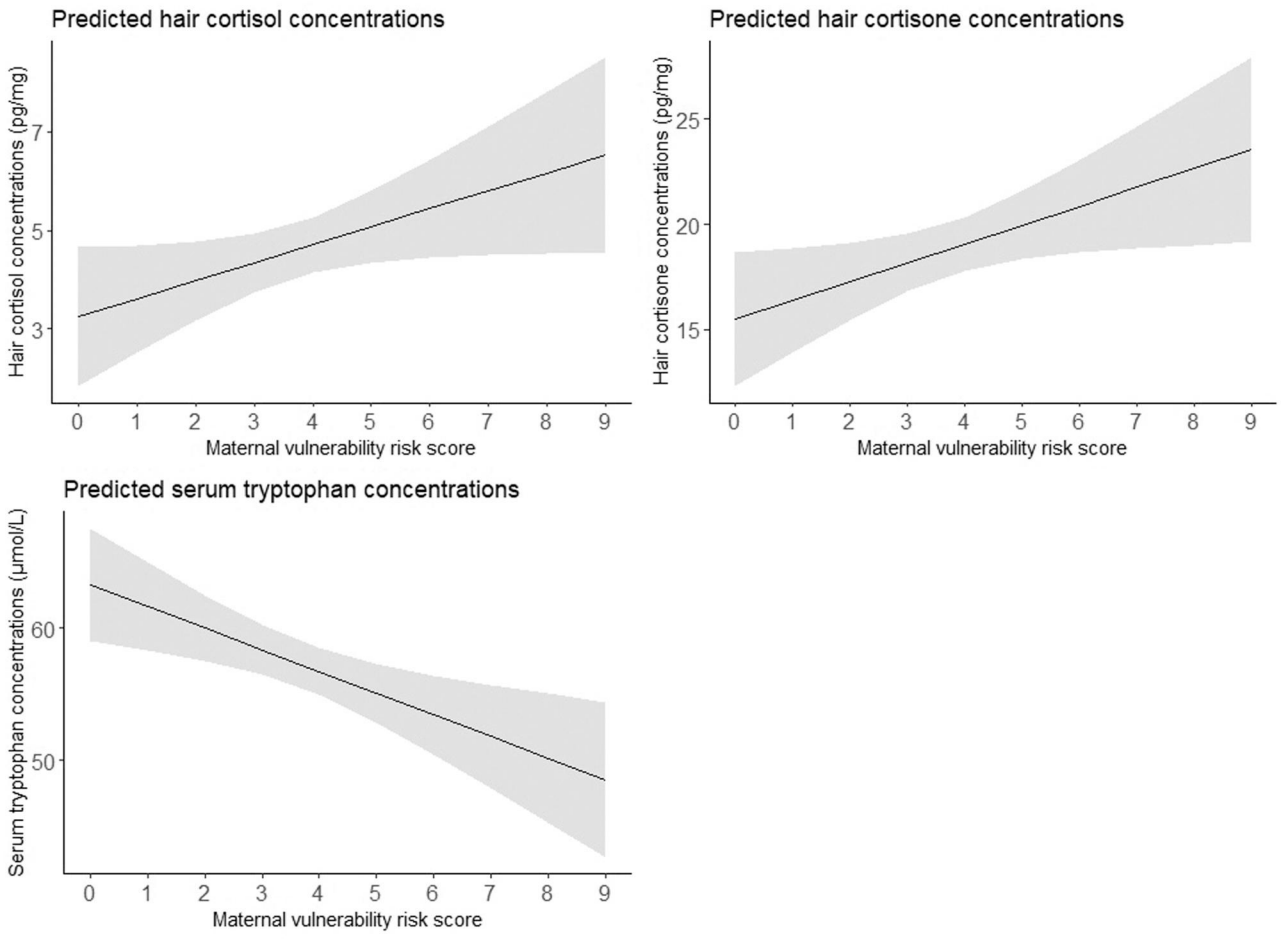
**Effect plots of associations between the maternal vulnerability risk score and stress biomarkers.** The values presented in the effect plots are predicted values, which represent the estimated outcomes based on the multivariable linear regression model that explores the association between the maternal vulnerability risk score and stress biomarkers. In this figure, each panel represents the association between the maternal vulnerability risk score and a different biomarker. The models with hair cortisol or hair cortisone concentrations as outcome were adjusted for corticosteroid use within the last 3 months and natural hair color, and the model with tryptophan concentrations as outcome were adjusted for gestational age at blood sampling. The solid line represents the estimated regression line, while the shaded area represents the 95% CI around the regression line.

The associations between the maternal vulnerability risk score and stress biomarkers are shown in Table 2 and visualized in Fig. 3. A higher maternal vulnerability risk score was associated with increased hair cortisol and cortisone concentrations (β = 0.366, 95% CI = 0.010–0.722; β = 0.897, 95% CI = 0.102–1.691). Log-transformation of hair cortisol and cortisone concentrations and exclusion of cases with permed hair (n* *=* *1) revealed comparable results (Supplementary Table S6). In contrast, a higher maternal vulnerability risk score was associated with decreased tryptophan concentrations (β = –1.637, 95% CI = –2.693 to -0.582), also after adjustment for protein intake (Supplementary Table S6).

**Table 2.** Associations between the maternal vulnerability risk score and stress biomarkers.

Linear regression of metabolite concentrations on maternal vulnerability risk score (n* *=* *132)	Model 1			Model 2		
	β	95% CI	*P*-value	β	95% CI	*P*-value
**Biomarkers in hair**						
Cortisol (pg/mg)	**0.383**	**0.044–0.722**	**0.027**	**0.366**	**0.010–0.722**	**0.044**
Cortisone (pg/mg)	0.780	–0.015 to 1.575	0.055	**0.897**	**0.102–1.691**	**0.027**
**Biomarkers in blood**						
Tryptophan (µmol/L)	**–1.648**	**–2.698 to –0.598**	**0.002**	**–1.637**	**–2.693 to –0.582**	**0.003**
Kynurenine (µmol/L)	–0.031	–0.073 to 0.010	0.137	–0.029	–0.070 to 0.012	0.162
5-hydroxytryptophan (nmol/L)	–0.029	–0.189 to 0.130	0.715	–0.026	–0.186 to 0.134	0.748
5-hydroxytryptamine (nmol/L)	–28.453	–61.553 to 4.647	0.091	–27.789	–61.008 to 5.430	0.100
5-hydroxyindole acetic acid (nmol/L)	**–3.233**	**–6.467 to 0.001**	**0.050**	–3.189	–6.439 to 0.060	0.054
Total homocysteine (µmol/L)	–0.065	–0.254 to 0.124	0.499	–0.077	–0.259 to 0.104	0.400
C-reactive protein (mg/L)	–0.402	–1.045 to 0.240	0.217	–0.395	–1.035 to 0.245	0.224

Model 1 was unadjusted. Model 2 for the biomarkers in hair was adjusted for corticosteroid use within the last 3 months and natural hair color. Model 2 for the biomarkers in blood was adjusted for gestational age at blood sampling. Values are presented in bold where *P* ≤ 0.05.

### Stress hormones and first-trimester growth

**Figure 4.**
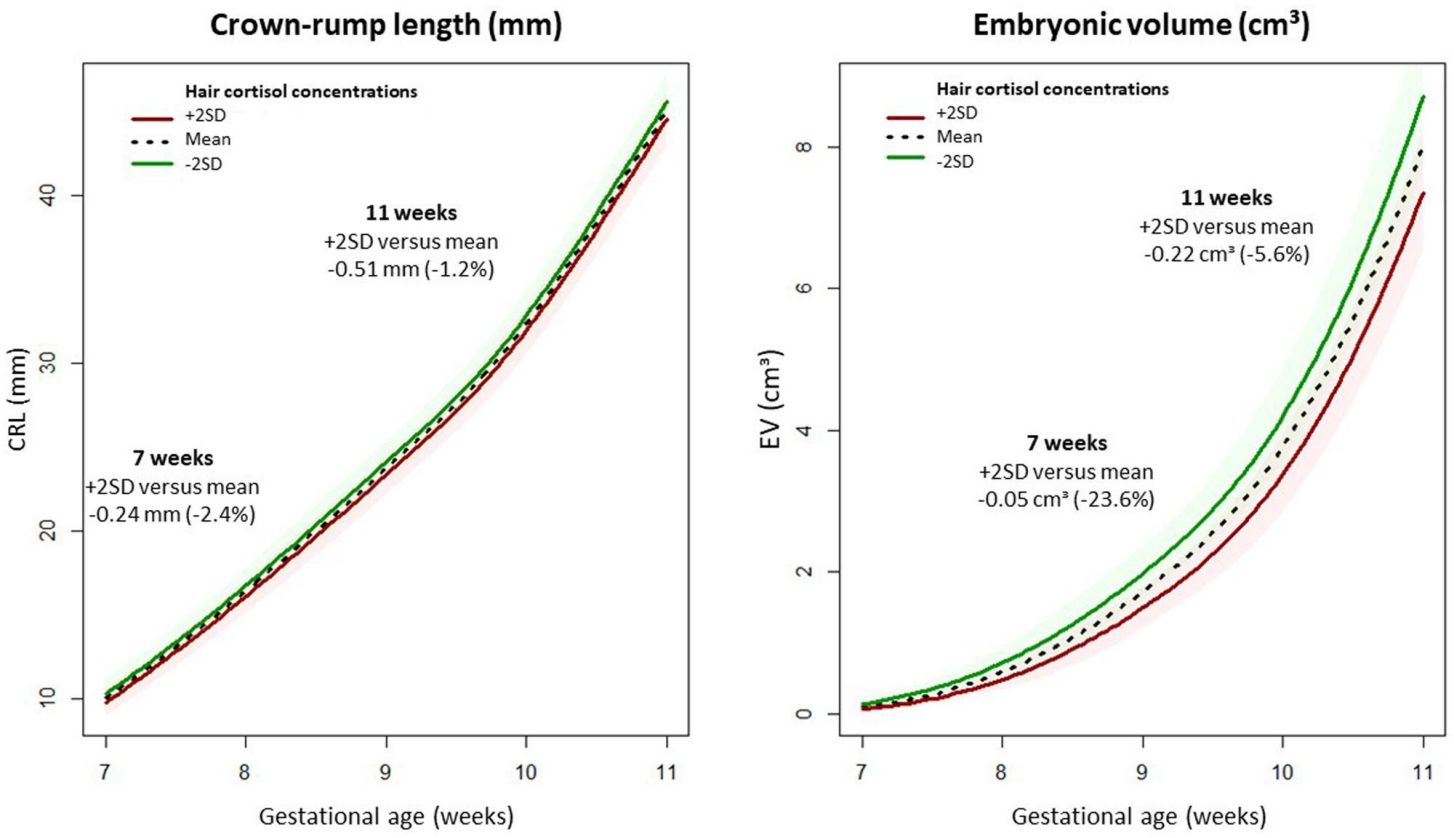
**Effect plots illustrating crown–rump length (CRL) and embryonic volume (EV) trajectories during the first trimester of pregnancy stratified for hair cortisol concentrations.** The effect plots visually depict the median CRL- and EV-trajectories during the first trimester of pregnancy predicted by multivariable mixed models for the mean hair cortisol concentration and concentrations 2SD, above and below the mean. In this figure, the left panel represents the CRL-trajectory and the right panel the EV-trajectory. The models were adjusted for corticosteroid use within the last 3 months, natural hair color, age, BMI, smoking, vegetable intake, and fetal sex. The solid line represents the estimated regression line, while the shaded area represents the 95% CI around the regression line.

The associations between hair cortisol and cortisone concentrations and CRL- and EV-trajectories are shown in Table 3. Hair cortisol concentrations were negatively associated with both CRL- and EV-trajectories. This negative association was only statistically significant for EV-trajectories (β = –0.010, 95% CI = –0.017 to –0.002). As an illustration, the median CRL- and EV-trajectories for women with +2SD, mean and –2SD hair cortisol concentrations are shown in Fig. 4. No associations were found between hair cortisone concentrations and CRL- or EV-trajectories.

**Table 3.** Associations between hair cortisol and cortisone concentrations and first-trimester growth.

Mixed models (n* *=* *107)	Model 1			Model 2		
	β	95% CI	*P*-value	β	95% CI	*P*-value
**Crown–rump length (√mm)**						
Cortisol (pg/mg)	–0.003	–0.016 to 0.010	0.620	–0.007	–0.019 to 0.006	0.312
Cortisone (pg/mg)	0.001	–0.004 to 0.008	0.555	0.001	–0.005 to 0.007	0.820
**Embryonic volume (^3^√cm^3^)**						
Cortisol (pg/mg)	–0.008	–0.016 to 0.000	0.057	**–0.010**	**–0.017 to –0.002**	**0.011**
Cortisone (pg/mg)	0.001	–0.003 to 0.004	0.767	0.000	–0.004 to 0.004	0.964

Model 1 was adjusted for gestational age at the 3D ultrasound scan visit. Model 2 was additionally adjusted for corticosteroid use within the last 3 months, natural hair color, age, BMI, smoking, vegetable intake, and fetal sex. Values are presented in bold where *P* ≤ 0.05.

The mediation analysis revealed no mediation by tryptophan metabolite concentrations in the relationship between hair cortisol concentrations and EV-trajectories (Supplementary Table S7).

Comparable results were found when hair cortisol and cortisone were log-transformed and when cases with permed hair were excluded (n* *=* *1) (Supplementary Table S8).

## Discussion

### Principal findings

This study showed that a higher maternal vulnerability risk score during the periconception period was associated with elevated concentrations of stress hormones (hair cortisol and cortisone) and reduced tryptophan in the first trimester of pregnancy. Higher hair cortisol concentrations in the first trimester of pregnancy were associated with reduced EV, with no mediation observed by tryptophan. No associations were found between first-trimester hair cortisone concentrations and first-trimester growth.

### Comparison with previous studies and interpretation

#### Stress biomarkers and maternal vulnerability

To the best of our knowledge, this is the first study that identified potential biological pathways involved in maternal vulnerability. In line with our hypothesis, a higher maternal vulnerability risk score was associated with increased stress hormone concentrations in hair. This implies that exposure to an accumulation of these risk factors induces a chronic stress response, resulting in long-term exposure to stress hormones. In addition, a higher maternal vulnerability risk score was associated with reduced tryptophan concentrations, independent of protein intake, suggesting an altered tryptophan metabolism in highly vulnerable women. Contrary to our expectations, stress hormones and tryptophan metabolites showed no correlation, indicating that vulnerability impacts both of these biological processes with minimal mutual influence.

No associations were found between the maternal vulnerability risk score and CRP or total homocysteine concentrations, which could be explained by the low concentrations and narrow ranges of these biomarkers in our study (CRP: two-thirds <5 mg/L, IQR = 2.1–6.3; total homocysteine: all <15 µmol/L, IQR = 5.5–7.2). Future research investigating the association between maternal vulnerability and inflammation should use a broader panel of immune markers, such as cytokines that are considered more specific inflammatory biomarkers than CRP (Coussons-Read *et al.*, 2007). Most women started folic acid supplement use before conception (85.6%), an important determinant of the one-carbon metabolism, which may account for the observed low total homocysteine concentrations (Steegers-Theunissen *et al.*, 2013).

#### Stress hormones and first-trimester growth

Previous research on stress hormones during pregnancy has mainly focused on stress hormones in saliva and blood, which can be influenced by circadian rhythm and acute stressors (Meyer and Novak, 2012; Lee *et al.*, 2015; Noppe *et al.*, 2015). Studies on stress hormones in hair, reflecting long-term exposure to elevated levels of stress hormones, and pregnancy course and outcome are scarce and inconsistent. In a recent study, a negative association was found between hair cortisol concentrations and birthweight of male newborns, while another study found no associations (Flom *et al.*, 2018; Conradt *et al.*, 2020). No study in humans has investigated the impact of long-term exposure to stress hormones on first-trimester growth yet.

Our results showed that exposure to elevated cortisol concentrations during the first trimester of pregnancy, as reflected by high hair cortisol concentrations, is associated with reduced EV. No mediation by tryptophan on the association between hair cortisol concentrations and first-trimester growth was found. Thus, our findings suggest that first-trimester maternal cortisol concentrations affect first-trimester growth via other mechanisms than via degradation of tryptophan. This corresponds with our recent finding that tryptophan was not associated with first-trimester growth (van Zundert *et al.*, 2024).

During early pregnancy, glucocorticoids are required for embryonic and fetal growth and development because of their involvement in cell differentiation and maturation of tissues and organ systems (Cottrell and Seckl, 2009). Conversely, excess of glucocorticoids can damage gametes, disrupt the hypothalamic–adrenal–gonadal axis, and impact uterine environment, thereby influencing implantation and embryonic and placental development (Joseph and Whirledge, 2017). In addition, there is mounting evidence that excess of glucocorticoids can reduce embryonic growth and program non-communicable diseases later in life through epigenetic mechanisms, such as DNA methylation (Seckl, 2004; Crudo *et al.*, 2012; Moisiadis *et al.*, 2017).

Growing evidence suggests that during early pregnancy (<8 weeks gestation), maternal glucocorticoids reach the embryo via uterine gland secretions, which are taken up by the trophoblast and diffuse in the coelomic fluid, where they are absorbed by the yolk sac. There is a transition to hemotrophic nutrition with exchange between the maternal and fetal circulation at the start of the second trimester (Burton *et al.*, 2001). Under physiological conditions, the enzyme 11β-hydroxysteroid dehydrogenase 2 in the trophoblast regulates the interconversion of cortisol and cortisone (Arcuri *et al.*, 1998). However, when maternal cortisol concentrations are elevated, maternal cortisol can cross the placenta and enter the fetal circulation (Goedhart *et al.*, 2010).

### Strengths and limitations

The main strength of this study was its longitudinal prospective design that allowed for the collection of multiple data on various maternal characteristics that were used for the development of the maternal vulnerability risk score and were included as covariates in our models to minimize confounding bias. Although we could not exclude residual confounding from unknown or unobserved factors, the consistency in the direction of the associations in the models and in all sensitivity analyses suggests that our findings are unlikely to be influenced by residual confounding. As the study was performed in a tertiary hospital, the internal validity was high, but the external validity has to be further investigated. Another strength was the use of reliable and reproducible first-trimester growth measurements using an AI algorithm and VR techniques (Rousian *et al.*, 2010; Rousian *et al.*, 2021; Bastiaansen *et al.*, 2023). Furthermore, we used validated LC-MS/MS methods for determination of stress hormones in scalp hair, which reflect long-term exposure, and is less influenced by circadian rhythm and acute stressors than circulating concentrations (Meyer and Novak, 2012; Lee *et al.*, 2015; Noppe *et al.*, 2015; Mirzaian *et al.*, 2023). However, several methodological considerations must be taken into account for using scalp hair, especially during pregnancy (Marceau *et al.*, 2020). Variability in hair growth rates and changes in hair diameter during pregnancy could have biased our results. Moreover, proper washing of hair samples is essential to avoid contamination from sweat or sebum. In our study, we addressed this by performing multiple wash steps on hair samples to remove residual cortisol from sweat or sebum (Mirzaian *et al.*, 2023). While self-reported data are an inevitable aspect of cohort studies, it can introduce bias, especially when dealing with sensitive topics such as smoking, alcohol use, and drug use during pregnancy (Ondersma *et al.*, 2019). Women may underreport these behaviors, which could lead to bias in our data collection, potentially affecting the internal validity of our results. The degree of maternal vulnerability was based on the number of risk factors without a weight to which women were exposed, in accordance with our previous study (van Zundert *et al.*, 2022). To develop a weighted maternal vulnerability risk score, more data on effect sizes and the role of social protective factors are needed. Additionally, the consistency and reliability of this new risk score should be evaluated. Finally, potential confounders were identified through a data-driven approach using bivariable analyses. Given the relatively small sample size, the results of these analyses (Supplementary Tables S3 and S4) should be interpreted with caution.

### Implications and future research

The current study addressed a research gap regarding the biological pathways involved in maternal vulnerability. Our results suggest that the HPA axis is chronically activated, and the tryptophan metabolism is altered in women with a high degree of maternal vulnerability. This study also provided evidence suggesting that exposure to stress hormones during the first trimester of pregnancy may be associated with reduced growth of the embryo. Hence, stress hormones may be a mediating factor in the association between maternal vulnerability and reduced first-trimester growth. These findings underscore the importance of identifying women with a high degree of vulnerability as early as possible, and at least before conception, to optimize first-trimester growth, and subsequent health outcomes. Given the complex nature of maternal vulnerability, it is likely that the effects of maternal vulnerability on pregnancy outcomes are influenced by multiple interacting biological pathways. Future research is needed to validate our findings and to explore additional underlying pathways of maternal vulnerability, such as other inflammatory and immune pathways, to identify a biological signature for maternal vulnerability. This could create opportunities for prediction, prevention, and personalized medicine.

## Conclusion

In conclusion, this study showed that a higher degree of maternal vulnerability during the periconception period is associated with higher stress hormone concentrations and lower tryptophan concentrations. In addition, our study shows that exposure to increased cortisol concentrations during the first trimester of pregnancy has a negative impact on first-trimester growth. This implies that the chronic stress due to exposure to prolonged or repeated exposure to vulnerability markers, at least partly, explains the negative association between maternal vulnerability and impaired first-trimester growth.

## Supplementary Material

deae211_Supplementary_Figure_S1

deae211_Supplementary_Figure_S2

deae211_Supplementary_Table_S1

deae211_Supplementary_Table_S2

deae211_Supplementary_Table_S3

deae211_Supplementary_Table_S4

deae211_Supplementary_Table_S5

deae211_Supplementary_Table_S6

deae211_Supplementary_Table_S7

deae211_Supplementary_Table_S8

## Data Availability

The data underlying this article will be shared upon reasonable request to the corresponding author.
