# Supplementary material for: The impact of maternal vulnerability on stress biomarkers and first-trimester growth: the Rotterdam Periconceptional Cohort (Predict Study)
Source: Hum Reprod. 2024 Sep 19;39(11):2423–33. doi: 10.1093/humrep/deae211 (PMC11532602; doi:10.1093/humrep/deae211)
Supplement: deae211_Supplementary_Figure_S1 [file deae211_supplementary_figure_s1.pdf]

Human Reproduction, 2024, 1–1
https://doi.org/10.1093/humrep/deae211
Supplementary Data

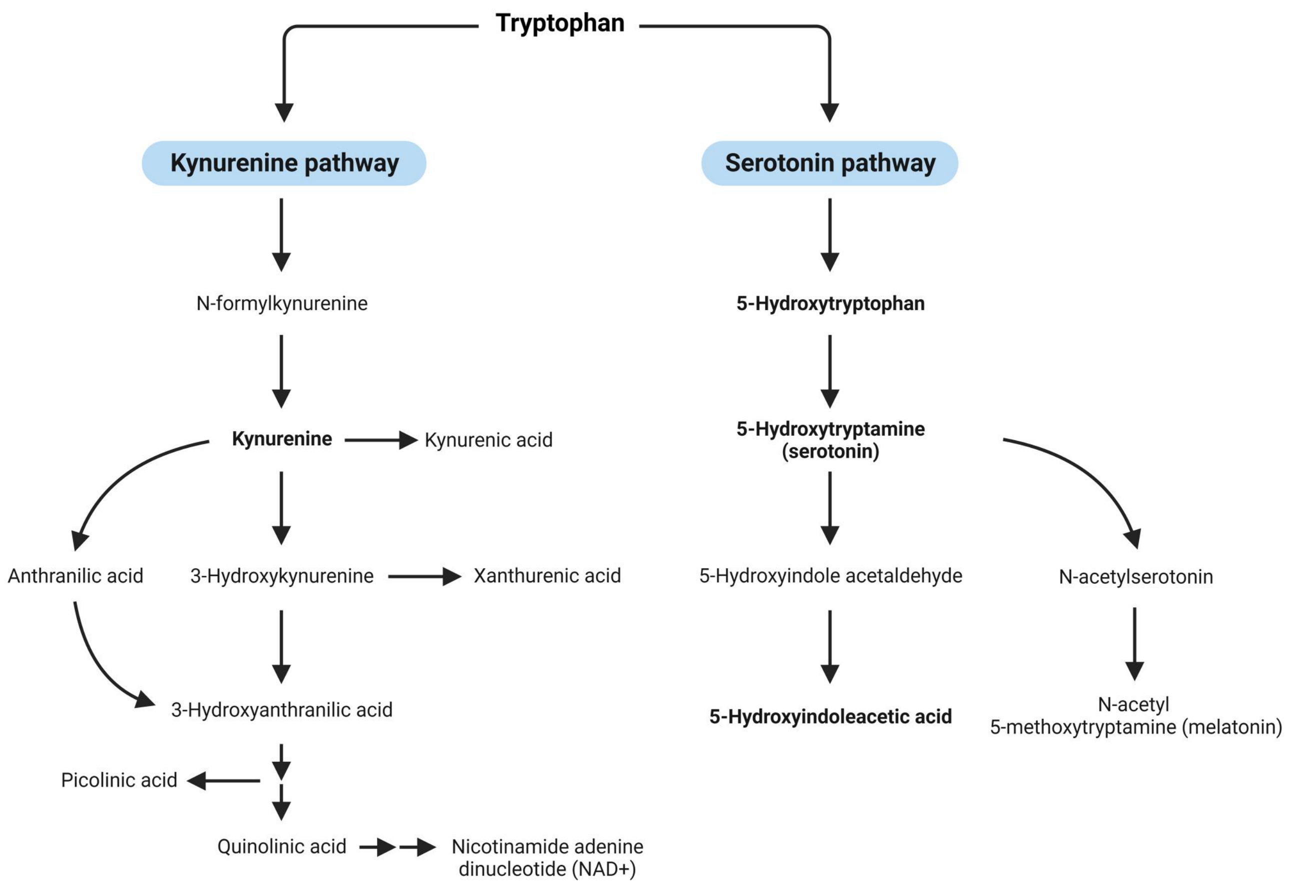

Supplementary Figure S1. The kynurenine and serotonin pathways of tryptophan metabolism. The metabolites measured in this study are highlighted in bold. Tryptophan is converted to N-formylkynurenine by hepatic tryptophan 2,3-dioxygenase (TDO) or extra-hepatic indoleamine 2,3-dioxygenase (IDO). This step serves as the rate-limiting step in the kynurenine pathway. N-formylkynurenine is then hydrolyzed to kynurenine by anthranilic acid, quinolinic acid, picolinic acid, and nicotinamide adenine dinucleotide (NADþ). In the serotonin pathway, tryptophan is first converted to 5-hydroxytryptophan by tryptophan hydroxylase (TPH), which is the rate-limiting step in this pathway. Next, 5-hydroxytryptophan is converted into 5-hydroxytryptamine (serotonin) by aromatic amino acid decarboxylase. 5-Hydroxytryptamine is metabolized into 5-hydroxyindoleacetic acid through two enzymatic steps involving monoamine oxidase and aldehyde dehydrogenase. Serotonin converts to melatonin by two step reaction. First converts to N-acetylserotonin by serotonin N-acetyltransferase (SNAT) and then to melatonin by N-Acetylserotonin O-methyltransferase (ASMT).
