# Supplementary material for: The impact of maternal vulnerability on stress biomarkers and first-trimester growth: the Rotterdam Periconceptional Cohort (Predict Study)
Source: Hum Reprod. 2024 Sep 19;39(11):2423–33. doi: 10.1093/humrep/deae211 (PMC11532602; doi:10.1093/humrep/deae211)
Supplement: deae211_Supplementary_Figure_S2 [file deae211_supplementary_figure_s2.pdf]

OXFORD
human reproduction

*Human Reproduction*, 2024, 1–1
https://doi.org/10.1093/humrep/deae211
Supplementary Data

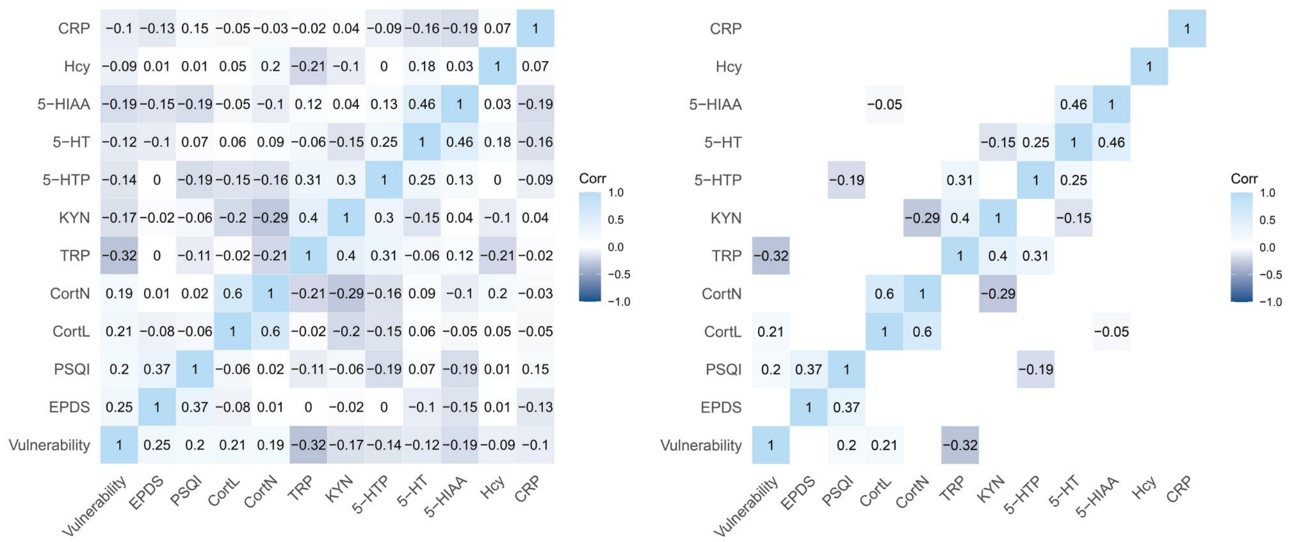


**Supplementary Figure S2. Correlation matrices of maternal vulnerability, mental distress, and stress biomarkers.** Spearman's correlations were used to estimate the correlations. The left correlation matrix includes all correlations, while the right correlation matrix includes only the statistically significant correlations. EPDS, Edinburgh Postnatal (Postpartum) Depression Scale; PSQI, Pittsburgh Sleep Quality Index; CortL, cortisol; CortN, cortisone; TRP, tryptophan; KYN, kynurenine, 5-HTP, 5-hydroxytryptophan; 5-HT, 5-hydroxytryptamine; 5-HIAA, 5-hydroxyindoleacetic acid; Hcy, total homocysteine; CRP, C-reactive protein.
