# Supplementary material for: The impact of maternal vulnerability on stress biomarkers and first-trimester growth: the Rotterdam Periconceptional Cohort (Predict Study)
Source: Hum Reprod. 2024 Sep 19;39(11):2423–33. doi: 10.1093/humrep/deae211 (PMC11532602; doi:10.1093/humrep/deae211)
Supplement: deae211_Supplementary_Table_S1 [file deae211_supplementary_table_s1.pdf]

OXFORD
human reproduction

Human Reproduction, 2024, 1–1
https://doi.org/10.1093/humrep/deae211
Supplementary Data

Supplementary Table S1. Summary of the stress biomarker distribution and quantification of the study samples.

Stress biomarkers	Distribution (median (IQR))	LLQ	LLD	≥LLQ (n (%))
Hair cortisol (pg/mg)	3.8 (2.7, 5.0)	1.0		127 (100%)
Missing				5
Hair cortisone (pg/mg)	17.1 (13.1, 22.8)	1.5		129 (100%)
Missing				3
Tryptophan (μmol/L)	56.0 (51.2, 63.0)	0.043		132 (100%)
Missing				0
Kynurenine (μmol/L)	1.4 (1.1, 1.6)	0.011		132 (100%)
Missing				0
5-Hydroxytryptophan (nmol/L)	5.7 (5.0, 6.6)	4.0	2.6	121 (96%)
Missing				2
5-Hydroxytryptamine (nmol/L)	671.5 (519.5, 891.9)	22.0		132 (100%)
Missing				0
5-Hydroxyindoleacetic acid (nmol/L)	46.0 (38.2, 57.7)	8.0	4.0	132 (100%)
Missing				0
Total homocysteine (μmol/L)	6.0 (5.5, 7.2)	0.2		116 (100%)
Missing				16
C-reactive protein (mg/L)	3.4 (2.1, 6.3)	0.6		126 (95%)
Missing				0

IQR, interquartile ranges; LLQ, lower limit of quantitation; LLD, lower limit of detection.
