# Supplementary material for: The impact of maternal vulnerability on stress biomarkers and first-trimester growth: the Rotterdam Periconceptional Cohort (Predict Study)
Source: Hum Reprod. 2024 Sep 19;39(11):2423–33. doi: 10.1093/humrep/deae211 (PMC11532602; doi:10.1093/humrep/deae211)
Supplement: deae211_Supplementary_Table_S2 [file deae211_supplementary_table_s2.pdf]

OXFORD human reproduction

Human Reproduction, 2024, 1–1
https://doi.org/10.1093/humrep/deae211
Supplementary Data

Supplementary Table S2. Definition of vulnerability markers.

Domain	Vulnerability marker	Definition
Social	Young or advanced age at conception	<20 or ≥40 years
	Non-western geographical origin	African, Asian (except for Indonesia and Japan), Latin-American, and Turkish origin (Alders, 2001)[a]
	Single	No stable relationship
	Deprived neighborhood	A socioeconomic status score for neighborhoods <–0.1 as calculated by Statistics Netherlands based on welfare, level of education, and recent labor participation (Statistics Netherlands, 2022)
	Low level of education	International Standard Classification of Education (ISCED) 0 (early childhood education), ISCED 1 (primary education), and ISCED 2 (lower secondary education) (United Nations Educational Scientific and Cultural Organization, 2012)
Lifestyle	Smoking	Any smoking during the periconception period
	Alcohol consumption	Any alcohol consumption during the periconception period
	Drug use	Any drug use during the periconception period
	Inadequate fruit and vegetable intake	Eating <2 pieces of fruit a day or eating vegetables <7 days a week
	Inadequate physical activity	<150 min of physical activity per week (World Health Organization, 2010)
Medical	Chronic disease	Any inflammatory or cardiovascular disease
	Medication use	Any medication use during the periconception period, including prescribed and over-the-counter medication (WHO Collaborating Centre for Drug Statistics Methodology, 2020)
	Underweight or obesity	BMI <18.5 or ≥30 kg/m^2 (World Health Organization, 2000)
	Psychiatric disorders	Anxiety or depressive disorders according to the Diagnostic and Statistical Manual of Mental Disorders Fifth Edition (DSM-V) (American Psychiatric Association, 2013)

[a] Women with an Indonesian or Japanese origin were considered Western based on their socioeconomic status within the Dutch society.
