# Supplementary material for: The impact of maternal vulnerability on stress biomarkers and first-trimester growth: the Rotterdam Periconceptional Cohort (Predict Study)
Source: Hum Reprod. 2024 Sep 19;39(11):2423–33. doi: 10.1093/humrep/deae211 (PMC11532602; doi:10.1093/humrep/deae211)
Supplement: deae211_Supplementary_Table_S3 [file deae211_supplementary_table_s3.pdf]

OXFORD
human reproduction
Human Reproduction, 2024, 1–1
https://doi.org/10.1093/humrep/deae211
Supplementary Data

Supplementary Table S3. Associations between maternal hair and general characteristics and stress biomarkers.

	Cortisol (pg/mg)			Cortisone (pg/mg)		
Linear regression (n = 132)	β	95% CI	P-value	β	95% CI	P-value
**Hair characteristics**						
**Natural hair color (ref = black)**						
Brown	–0.596	–2.206 to 1.014	0.465	0.885	–2.919 to 4.689	0.646
Blond	**–1.650**	**–3.285 to –0.016**	**0.048**	–3.776	–7.629 to 0.078	0.055
Red	–3.406	–7.683 to 0.871	0.118	–4.199	–14.510 to 6.112	0.422
**Wash frequency (ref = <1 per week)**						
1–2 per week	–0.386	–2.705 to 1.933	0.742	1.377	–4.126 to 6.880	0.621
3–4 per week	–0.446	–2.868 to 1.977	0.716	–1.132	–6.870 to 4.605	0.697
>4 per week	0.151	–2.527 to 2.829	0.911	6.001	–0.358 to 12.359	0.064
**Most recent hair wash (ref = <24 h ago)**						
24–48 h ago	–0.148	–1.422 to 1.127	0.819	–0.474	–3.587 to 2.640	0.764
>48 h ago	0.806	–0.423 to 2.035	0.197	1.838	–1.187 to 4.863	0.231
**Regularly sweating on head**	0.450	–1.250 to 2.151	0.601	–0.888	–4.957 to 3.180	0.666
**Dandruff (ref = no)**						
Yes, anti-dandruff shampoo use	–1.057	–2.469 to 0.355	0.141	–2.095	–5.604 to 1.414	0.240
Yes, no anti-dandruff shampoo use	–1.310	–3.734 to 1.114	0.287	–0.840	–6.872 to 5.191	0.783
**Hair product use (ref = no)**						
Yes, mousse	0.896	–2.509 to 4.300	0.603	0.792	–7.814 to 9.398	0.856
Yes, gel	2635	–0.039 to 5.309	0.055	0.172	–6.587 to 6.931	0.960
Yes, wax	1012	–1.957 to 3.981	0.501	0.905	–6.601 to 8.410	0.812
Yes, hairspray	–0.216	–2.172 to 1.740	0.827	–0.909	–5.850 to 4.032	0.716
Yes, other	0.927	–0.245 to 2.098	0.120	0.768	–2.163 to 3.698	0.605
**Hair bleached**	–0.439	–2.140 to 1.261	0.610	0.983	–3.221 to 5.187	0.644
**Hair dyed**	0.219	–1.144 to 1.582	0.751	1.746	–1.607 to 5.100	0.305
**Hair permed**	**7.720**	**2.043–13.397**	**0.008**	**37.384**	**24.521–50.246**	**<0.001**
**Hair straightened**	0.934	–2.016 to 3.885	0.532	–0.779	–8.084 to 6.526	0.833
**Corticosteroid use**	–0.468	–2.065 to 1.129	0.563	**–3.810**	**–7.704** to **0.083**	**0.054**
**Weight of the hair sample (mg)**	–0.143	–0.369 to 0.083	0.214	–0.147	–0.697 to 0.403	0.598
**Gestational age at sampling**	–0.227	–0.631 to 0.176	0.267	0.032	–0.948 to 1.012	0.948
**General characteristics**						
**Age (years)**	–0.001	–0.125 to 0.124	0.994	0.214	–0.091 to 0.518	0.168
**Parity (nulliparous versus multiparous)**	–0.336	–1.381 to 0.709	0.526	–0.768	–3.338 to 1.803	0.556
**BMI (kg/m^2)**	0.018	–0.100 to 0.137	0.760	0.033	–0.256 to 0.323	0.820
**Geographical origin (non-Western versus Western)**	0.431	–0.957 to 1.818	0.540	1.052	–2.374 to 4.478	0.544
**Educational level**	0.804	–1.439 to 3.048	0.479	2.577	–2.941 to 8.094	0.357
Low versus medium						
High versus medium	0.062	–1.050 to 1.174	0.912	1.038	–1.667 to 3.743	0.449
**Smoking (yes versus no)**	–0.610	–2.371 to 1.151	0.494	–0.077	–4.437 to 4.283	0.972
**Alcohol consumption (yes versus no)**	–0.573	–1.687 to 0.541	0.311	–0.359	–3.116 to 2.398	0.797
**Drug use (yes versus no)**	0.891	–2.503 to 4.286	0.604	1.589	–6.810 to 9.988	0.709
**Fruit intake (inadequate versus adequate)**	0.593	–0.571 to 1.757	0.315	2.523	–0.222 to 5.268	0.071
**Vegetable intake (inadequate versus adequate)**	0.470	–0.660 to 1.600	0.411	2.052	–0.616 to 4.721	0.130
**Folic acid supplement use (inadequate versus adequate)**	–0.447	–1.924 to 1.031	0.551	0.351	–3.222 to 3.924	0.846
**Mode of conception (IVF/ICSI versus natural**	0.379	–0.660 to 1.419	0.472	–1.228	–3.774 to 1.318	0.342
**Fetal sex (girl versus boy)**	0.923	–0.125 to 1.970	0.084	0.506	–2.050 to 3.063	0.696

Values are presented in bold where $P \leq 0.05$.
