# Supplementary material for: The impact of maternal vulnerability on stress biomarkers and first-trimester growth: the Rotterdam Periconceptional Cohort (Predict Study)
Source: Hum Reprod. 2024 Sep 19;39(11):2423–33. doi: 10.1093/humrep/deae211 (PMC11532602; doi:10.1093/humrep/deae211)
Supplement: deae211_Supplementary_Table_S4 [file deae211_supplementary_table_s4.pdf]

Human Reproduction, 2024, 1–1
https://doi.org/10.1093/humrep/deae211
Supplementary Data

Supplementary Table S4. Associations between maternal general characteristics and first-trimester growth parameters.

	Crown–rump length ($\sqrt{mm}$)			Embryonic volume ($\sqrt[3]{cm^3}$)		
**Mixed models (n = 107)**	**β**	**95% CI**	**P-value**	**β**	**95% CI**	**P-value**
**General characteristics**						
Age (years)	**−0.009**	**−0.019 to 0.000**	**0.054**	−0.005	−0.011 to 0.001	0.131
Parity (nulliparous versus multiparous)	0.051	−0.037 to 0.139	0.257	0.041	−0.014 to 0.096	0.139
BMI (kg/m^2)	−0.010	−0.021 to 0.001	0.070	**−0.007**	**−0.013 to 0.0001**	**0.048**
Geographical origin (Non-Western versus Western)	−0.028	−0.159 to 0.103	0.672	−0.044	−0.129 to 0.039	0.293
Educational level (low versus medium)	−0.047	−0.209 to 0.116	0.571	−0.034	−0.138 to 0.069	0.511
Educational level (high versus medium)	−0.022	−0.117 to 0.072	0.641	−0.021	−0.021 to 0.038	0.483
Smoking (yes versus no)	0.090	−0.048 to 0.227	0.199	**0.088**	**0.002–0.175**	**0.045**
Alcohol consumption (yes versus no)	0.084	−0.005 to 0.172	0.063	0.039	−0.019 to 0.096	0.185
Drug use (yes versus no)	0.181	−0.083 to 0.445	0.176	0.045	−0.119 to 0.210	0.586
Fruit intake (inadequate versus adequate)	0.037	−0.048 to 0.123	0.389	0.018	−0.038 to 0.075	0.532
Vegetable intake (inadequate versus adequate)	0.048	−0.036 to 0.131	0.258	**0.057**	**0.004–0.112**	**0.036**
Folic acid supplement use (inadequate versus adequate)	0.112	−0.043 to 0.267	0.156	0.055	−0.038 to 0.148	0.245
Mode of conception (IVF/ICSI versus natural)	0.032	−0.063 to 0.127	0.510	0.045	−0.014 to 0.104	0.131
Fetal sex (girl versus boy)	**−0.092**	**−0.177 to −0.006**	**0.036**	−0.033	−0.086 to 0.019	0.214

The model was adjusted for gestational age at the 3D ultrasound scan visit, BMI, IVF, and ICSI. Values are presented in bold where $P \leq 0.05$.
