# Supplementary material for: The impact of maternal vulnerability on stress biomarkers and first-trimester growth: the Rotterdam Periconceptional Cohort (Predict Study)
Source: Hum Reprod. 2024 Sep 19;39(11):2423–33. doi: 10.1093/humrep/deae211 (PMC11532602; doi:10.1093/humrep/deae211)
Supplement: deae211_Supplementary_Table_S5 [file deae211_supplementary_table_s5.pdf]

OXFORD
human reproduction
Human Reproduction, 2024, 1–1
https://doi.org/10.1093/humrep/deae211
Supplementary Data

Supplementary Table S5. Hair characteristics of the total study population.

Hair characteristics	n = 132
**Natural hair color**	**n (%)**
Black	18 (13.6%)
Brown	59 (44.7%)
Blond	53 (40.2%)
Red	2 (1.5%)
Missing	0
**Wash frequency**	
<1 time/week	8 (6.1%)
1–2 times/week	72 (54.5%)
3–4 times/week	36 (27.3%)
>4 times/week	16 (12.1%)
Missing	0
**Last hair wash**	
<24 h ago	51 (38.9%)
24–48 h ago	38 (29.0%)
> 48 h ago	42 (32.1%)
Missing	1
**Regularly sweating on head**	
Yes	15 (11.4%)
No	117 (88.6%)
Missing	0
**Dandruff**	
Yes, anti-dandruff shampoo use	7 (5.30%)
Yes, no anti-dandruff shampoo use	21 (15.9%)
No	104 (78.8%)
Missing	0
**Hair product use**	
Yes, mousse	3 (2.3%)
Yes, gel	5 (3.8%)
Yes, wax	4 (3.0%)
Yes, hairspray	10 (7.6%)
Yes, other	40 (30.3%)
No	70 (53.0%)
Missing	0
**Bleached hair**[a]	
Yes	14 (10.6%)
No	118 (89.4%)
Missing	0
**Dyed hair**[a]	
Yes	22 (16.7%)
No	110 (83.3%)
Missing	0
**Permed hair**[a]	
Yes	1 (0.8%)
No	131 (99.2%)
Missing	0
**Straightened hair**[a]	
Yes	4 (3.0%)
No	128 (97.0%)
Missing	0
**Any corticosteroid use**[b]	
Yes	16 (12.1%)
No	116 (87.9%)
Missing	0

[a] Within the last 1–2 months.
[b] Within the last 3 months, including oral, cutaneous, and inhalation corticosteroid use. The categorical variables were presented as numbers with corresponding percentages.
