# Supplementary material for: The impact of maternal vulnerability on stress biomarkers and first-trimester growth: the Rotterdam Periconceptional Cohort (Predict Study)
Source: Hum Reprod. 2024 Sep 19;39(11):2423–33. doi: 10.1093/humrep/deae211 (PMC11532602; doi:10.1093/humrep/deae211)
Supplement: deae211_Supplementary_Table_S6 [file deae211_supplementary_table_s6.pdf]

OXFORD
human reproduction

Human Reproduction, 2024, 1–1
https://doi.org/10.1093/humrep/deae211
Supplementary Data

Supplementary Table S6. Sensitivity analysis of the associations between the maternal vulnerability risk score and stress biomarkers.

Linear regression of metabolite concentrations on maternal vulnerability risk score (n = 132)		β	95% CI	P-value
**Biomarkers in hair[a]**				
Cortisol (pg/mg)	Model 1	**0.383**	**0.044–0.722**	**0.027**
	Model 2	**0.366**	**0.010–0.722**	**0.044**
Natural log of cortisol (pg/mg)	Model 1	**0.082**	**0.024–0.140**	**0.006**
	Model 2	**0.080**	**0.021–0.138**	**0.008**
Cortisol without cases with permed hair (pg/mg)	Model 1	**0.375**	**0.044–0.706**	**0.027**
	Model 2	**0.351**	**0.003–0.699**	**0.048**
Cortisone (pg/mg)	Model 1	0.780	−0.015 to 1.575	0.055
	Model 2	**0.897**	**0.102–1.691**	**0.027**
Natural log of cortisone (pg/mg)	Model 1	0.040	−0.003 to 0.084	0.070
	Model 2	**0.051**	**0.007–0.094**	**0.023**
Cortisone without cases with permed hair (pg/mg)	Model 1	**0.745**	**0.047–1.442**	**0.037**
	Model 2	**0.826**	**0.127–1.525**	**0.021**
**Biomarkers in blood[b]**				
Tryptophan (μmol/L)	Model 1	**−1.648**	**−2.698 to −0.598**	**0.002**
	Model 2	**−1.637**	**−2.693 to −0.582**	**0.003**
	Model 3	**−1.852**	**−3.122 to −0.582**	**0.005**

Model 1 was unadjusted. Model 2 for the biomarkers in hair was adjusted for corticosteroid use within the last 3 months and natural hair color. Model 2 for the biomarkers in blood was adjusted for gestational age at blood sampling. Model 3 was adjusted for gestational age at blood sampling and protein intake (n = 81). Values are presented in bold where $P \leq 0.05$.
