# Supplementary material for: The impact of maternal vulnerability on stress biomarkers and first-trimester growth: the Rotterdam Periconceptional Cohort (Predict Study)
Source: Hum Reprod. 2024 Sep 19;39(11):2423–33. doi: 10.1093/humrep/deae211 (PMC11532602; doi:10.1093/humrep/deae211)
Supplement: deae211_Supplementary_Table_S7 [file deae211_supplementary_table_s7.pdf]

OXFORD
human reproduction

Human Reproduction, 2024, 1–1
https://doi.org/10.1093/humrep/deae211
Supplementary Data

Supplementary Table S7. Association between hair cortisol concentrations and embryonic volume embryonic volume (EV)-trajectories potentially mediated by tryptophan concentrations.

Association between hair cortisol and embryonic volume	β	95% CI
**Total association**	**–0.0095**	**–0.0168 to –0.0023**
**Direct pathway**	**–0.0096**	**–0.0170 to –0.0023**
**Indirect pathway**	0.0001	–0.0016 to 0.0015

Model 2 was adjusted for corticosteroid use within the last 3 months, natural hair color, age, BMI, smoking, vegetable intake, and fetal sex. Hair cortisol concentrations are in pg/mg. Values are presented in bold where $P \leq 0.05$.
