# Supplementary material for: The impact of maternal vulnerability on stress biomarkers and first-trimester growth: the Rotterdam Periconceptional Cohort (Predict Study)
Source: Hum Reprod. 2024 Sep 19;39(11):2423–33. doi: 10.1093/humrep/deae211 (PMC11532602; doi:10.1093/humrep/deae211)
Supplement: deae211_Supplementary_Table_S8 [file deae211_supplementary_table_s8.pdf]

OXFORD
human reproduction

Human Reproduction, 2024, 1–1
https://doi.org/10.1093/humrep/deae211
Supplementary Data

Supplementary Table S8. Sensitivity analysis of the associations between hair cortisol and cortisone concentrations and first-trimester growth.

	Model 1			Model 2		
**Mixed models**	**β**	**95% CI**	**P-value**	**β**	**95% CI**	**P-value**
**Crown–rump length (√mm)**						
**Cortisol**	–0.003	–0.016 to 0.010	0.620	–0.007	–0.019 to 0.006	0.312
Natural log of cortisol	–0.003	–0.079 to 0.073	0.935	–0.038	–0.121 to 0.045	0.364
Without cases with permed hair	–0.002	–0.015 to 0.011	0.768	–0.004	–0.018 to 0.008	0.468
**Cortisone**	0.001	–0.004 to 0.008	0.555	0.001	–0.005 to 0.007	0.820
Natural log of cortisone	0.036	–0.070 to 0.142	0.501	0.025	–0.093 to 0.144	0.670
Without cases with permed hair	0.004	–0.002 to 0.011	0.238	0.004	–0.004 to 0.011	0.321
**Embryonic volume ($\sqrt[3]{cm^3}$)**						
**Cortisol**	–0.008	–0.016 to 0.000	0.057	**–0.010**	**–0.017 to –0.002**	**0.011**
Natural log of cortisol	–0.035	–0.083 to 0.014	0.160	**–0.056**	**–0.104 to –0.008**	**0.022**
Without cases with permed hair	–0.008	–0.016 to 0.000	0.059	**–0.010**	**–0.017 to –0.002**	**0.012**
**Cortisone**	0.001	–0.003 to 0.004	0.767	0.000	–0.004 to 0.004	0.964
Natural log of cortisone	0.013	–0.056 to 0.083	0.706	0.001	–0.004 to 0.005	0.735
Without cases with permed hair	0.001	–0.003 to 0.006	0.577	–0.001	–0.005 to 0.003	0.669

Model 1 was unadjusted. Model 2 was adjusted for corticosteroid use within the last 3 months, natural hair color, age, BMI, smoking, vegetable intake, and fetal sex. Hair cortisol and cortisone concentrations are in pg/mg. Values are presented in bold where $P \leq 0.05$.

© The Author(s) 2024. Published by Oxford University Press on behalf of European Society of Human Reproduction and Embryology.
This is an Open Access article distributed under the terms of the Creative Commons Attribution License (https://creativecommons.org/licenses/by/4.0/), which permits unrestricted reuse, distribution, and reproduction in any medium, provided the original work is properly cited.
Human Reproduction, 2024, 00, 1–21
https://doi.org/10.1093/humrep/deae211
Original Article
